# Graph convolutional network approach applied to predict hourly bike-sharing demands considering spatial, temporal, and global effects

**DOI:** 10.1371/journal.pone.0220782

**Published:** 2019-09-16

**Authors:** Tae San Kim, Won Kyung Lee, So Young Sohn

**Affiliations:** Department of Industrial Engineering, Yonsei University, Shinchon-dong, Seoul, Republic of Korea; HEC Montréal, CANADA

## Abstract

Solving the supply–demand imbalance is the most crucial issue for stable implementation of a public bike-sharing system. This gap can be reduced by increasing the accuracy of demand prediction by considering spatial and temporal properties of bike demand. However, only a few attempts have been made to account for both features simultaneously. Therefore, we propose a prediction framework based on graph convolutional networks. Our framework reflects not only spatial dependencies among stations, but also various temporal patterns over different periods. Additionally, we consider the influence of global variables, such as weather and weekday/weekend to reflect non-station-level changes. We compare our framework to other baseline models using the data from Seoul’s bike-sharing system. Results show that our approach has better performance than existing prediction models.

## Introduction

Recently, demand for bike-sharing systems has increased globally, owing to greener and healthier living styles [1]. However, the supply–demand imbalance remains the biggest obstacle to operating an efficient bike-sharing system [2]. To reduce the gap between supply and demand, manual monitoring systems have been used to enable bike relocation by truck [3]. Because this method consumes enormous labor and time, the accurate forecasting of real-time demand has gained importance. In this paper, we propose a framework for forecasting hourly bike demand at the station level, which can reduce operating costs and time requirements for bike-sharing systems.

Bike data has both spatial and temporal properties [4]. To improve prediction accuracy, it is important to understand both characteristics [5]. First, spatial properties indicate dependencies among stations. The inter-station relationship can be represented by geographical distances among stations. Furthermore, close stations are more similar than distant stations, according to the first law of geography [6]. For instance, if one station does not have any available bikes, the user will most likely rent one at a nearby station. However, the relationship among stations is not merely established by local distance. Another connection can be construed in terms of usage patterns. The mobility pattern of bike usage can be identified by usage pattern and can be utilized for forecasting upcoming demand [7]. For example, we should expect greater usages between residential stations and commercial stations, especially during rush-hour commute. These two types of stations are considered closely related, despite their distance. Second, from the temporal perspective, demand is not only influenced by temporal proximity, but also by repetitive patterns over a constant period [8]. For example, daily and weekly patterns can be employed to forecast future demand. Thus, these various temporal properties are just as important as spatial properties.

Most recently, deep learning has been applied to bike demand forecasting. Among the many approaches, the convolutional neural network (CNN) is one of the popular models being used to solve real-time bike demand forecasting [6, 9]. However, as CNN extracts the local features considering relativeness of local architecture of image, they only reflect inter-station relationship by geographical distance [1]. In order to overcome this drawback of CNN, there has been an attempt to apply deep learning architecture to graph data structure [10–11]. Graph represents heterogenous pairwise relationship of nodes, so it can capture non-Euclidean properties among the nodes [10].

Graph convolutional neural network (GCN), state-of-the-art deep learning model in graph theorem, is also applied to predict bike usage by exploiting underlying spatial properties [1]. GCN is a neural network technique that works on graph structures composed of nodes and edges [10]. These graph structures can reflect various relations among stations, so that various spatial properties can be considered by GCN. However, in our best knowledge, there were no studies that considered various temporal patterns while taking advantage of the graph theory. Demand patterns repeat periodically, so it can provide important clues in predicting next demand. Thus, we construct a GCN-based prediction model which incorporates not only different spatial characteristics, but also various temporal patterns. Moreover, we consider the influence of global variables, such as weather and weekday/weekend, to consider non-station level changes. We verify our model using real-world bike data and compare it to other baseline models in a prediction task.

This paper is organized as follows. We review various studies related to the prediction of bike demand in Section 2. Then, we propose a GCN architecture reflecting various spatio–temporal properties and global variables in Section 3. We apply our architecture to the Seoul bike-sharing system, “ddareungi”, and compare the performance of our model to that of other prediction models in Section 4. Finally, in Section 5, conclusions and future works are summarized.

## Literature review

### Spatio–temporal properties

From simple linear regression to statistically complex methods, early-stage studies were designed to find the relationship between spatio–temporal variables and bike demand. Frade and Ribeiro [12] estimated the effects of weather conditions and temporal features. Differing influences of temperature, rainfall, and snow were explored as weather conditions, and patterns of day-of-week and monthly total demand were extracted as temporal features. Multiple regression models were used to capture the simultaneous influence of all variables. Thomas et al. [13] used a similar regression model as did Frade and Ribeiro [12], but they considered different weather sensitivity for cycling purposes. Depending on the location, the purpose of riding varies. Thus, the degree of sensitivity to weather will differ depending on personal riding purposes. For example, recreational purposes are more affected than utilitarian purposes. Thus, individual sensitivity to weather is reflected by bike demand.

However, there have been studies that used complicated and hierarchical methods to reflect complex relationship among spatio–temporal variables. Regue and Recker [14] used a gradient boosting machine (GBM), a machine-learning method that uses a decision tree structure that enables prediction of bike demand. Spatio–temporal variables include the number of bikes concurrently at a station, hourly weather conditions, holidays, and current time. The hierarchical relationship among these variables was captured with a tree structure. The results showed that the GBM showed better performance than simple linear regression models. Froehlich et al. [15] applied clustering techniques to group bike stations based on their frequency of usage. They performed a simple Bayesian network analysis using current time, prediction window, and the number of currently available bikes to predict *delta*, a continuous Gaussian variable that represents forecasted variation of demand in a prediction window. By adding the *delta* to the most resent observation, predictions were made. They also showed that stations in the same cluster had similar circumstances related to location, and each cluster had different patterns of hourly future usage. Thus, they demonstrated relationships among time-of-day, geographic location, and demand.

### Deep learning approaches

However, because bike demand is affected by many complex factors [9], it is difficult to capture all the underlying relations of demand solely with observable variables [16]. Deep learning can extract highly meaningful latent features from raw datasets. Recently, this has been regarded as one of the most powerful methods for obtaining more predictive power. In the domain of bike-demand prediction, deep learning models have been proposed [6, 9]. Zhang et al. [6] presented a CNN study that predicted bike demand using image data, showing demand based on regions of Beijing. CNN is a deep learning method used for extracting features focused on geographical connectivity among station locations [17]. As an image passes through the convolution layer, the neighboring local information is combined into one grid of image by localized filter [17]. Through the repetition of this procedure, highly meaningful local features are extracted. The authors built a CNN model that learned timely sequential images, so that geographical distance and temporal effects could be captured simultaneously. Thus, the prediction performance significantly improved. In a subsequent paper, Zhang et al. [9] showed enhanced results using residual neural network (ResNet), another state-of-the-art CNN architecture.

## Methodology

### Framework

**Fig 1:**
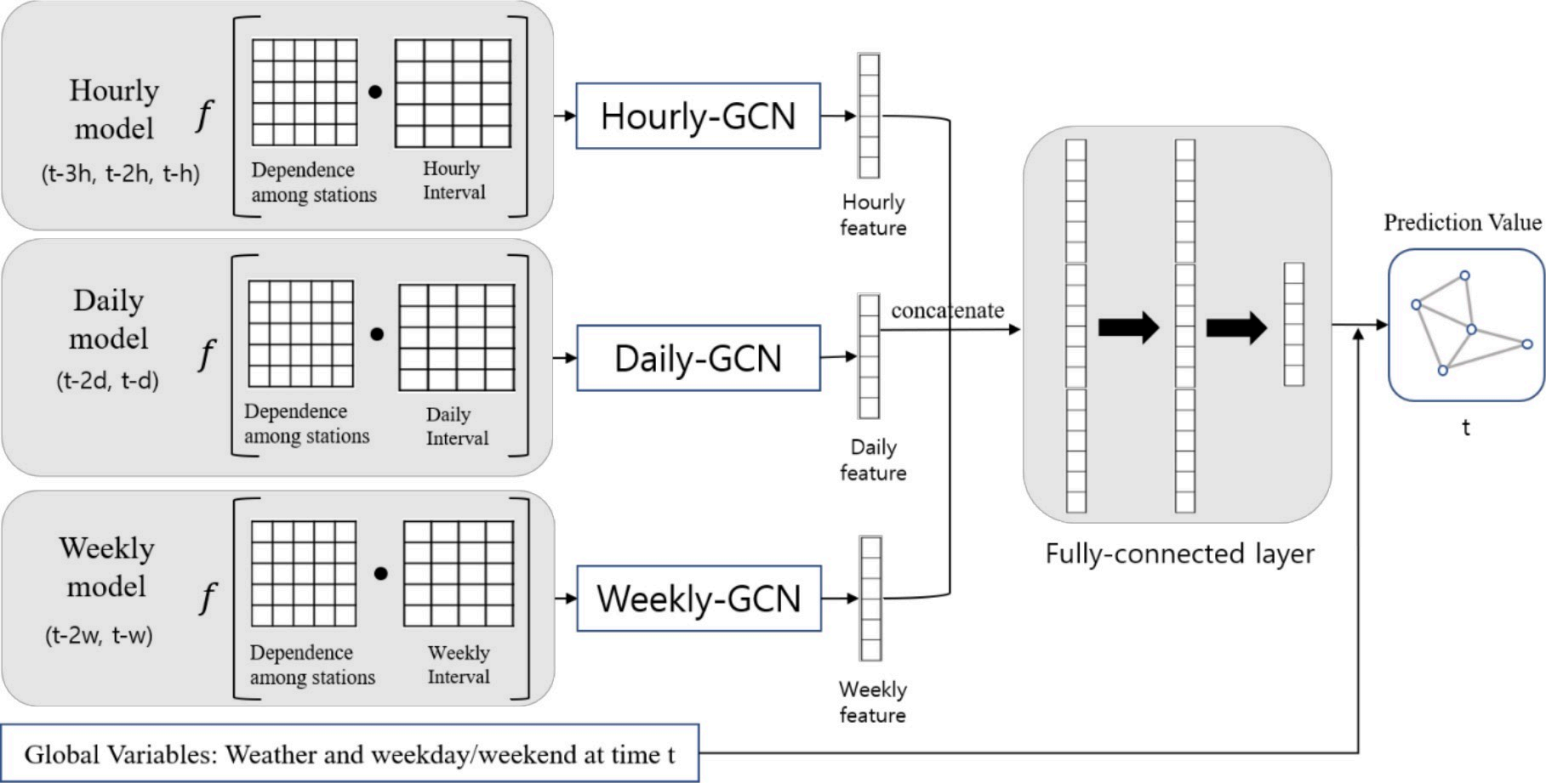
Framework of proposed GCN.

This paper presents a GCN based framework that reflects the influence of various spatio-temporal properties and global variables simultaneously. GCN is a neural network designed to work on a structured graph, instead of regular grids [10]. The graph structure consists of nodes and edges, so this paper defines nodes as stations, whereas edges as dependencies among stations. Fig 1 shows the detailed structure of our model. First, we define spatial and temporal properties for extracting a graph structure from a raw dataset. For spatial properties, we employ both the geographical distance-based graph and historical usage pattern-based graph. The performance of GCN relies on the relationship among stations [10]. Thus, we use the two graph structures separately and investigate which structure is better for demand prediction. For temporal properties, we construct hourly, daily, and weekly GCN models to capture the repeated patterns over time. For simultaneous training of these three types of temporal patterns, all features are concatenated and fused into a fully connected layer (FCL). Subsequently, global variables that can potentially affect bike demand, such as weather condition and different pattern of weekday and weekend, are considered. The details of our architecture are explained below.

### Graph convolutional networks

The reason for using GCN is to consider relationship among stations that can be represented in a graphical structure. Thus, to employ a GCN, the graph structure must first be extracted from the raw dataset. In this study, the graph is represented as G = (V,E,*x*_*i*_), where V is a set of bike stations of size *N*_*s*_, E is the set of edges between stations, and *x*_*i*_ is demand for every station *i* to predict demand at time *t*. In this study, we aimed to predict the next time bike demand of all stations, namely the corresponding target vector *y*_*t*_ *∈ R*^*Ns*^.

To conduct the graph convolutional process, we first converted the raw information to graph representations. The graph representations based on bike demand could be expressed in the form of an adjacency matrix and feature matrix. The adjacency matrix A∈*R*^*Ns*×*Ns*^ represents the network connection among nodes, whereas feature matrix H = [*X*^*t*-*W*^, …, *X*^*t*-1^] *∈ R*^*Ns*×*Nw*^, where *N*_*w*_ is the time window size that indicates how many previous demands are considered for every station, refers to the previous demand of each station related to target demand. We compose two types of A with the distances and usage patterns among stations and constructed three *H* composed of the sequential demand with different time intervals for the hourly, daily, and weekly GCN model, respectively. Since constructed *A* and *H* jointly predicted the next hour demand, the GCN model could capture spatial and temporal properties simultaneously.

**Fig 2:**
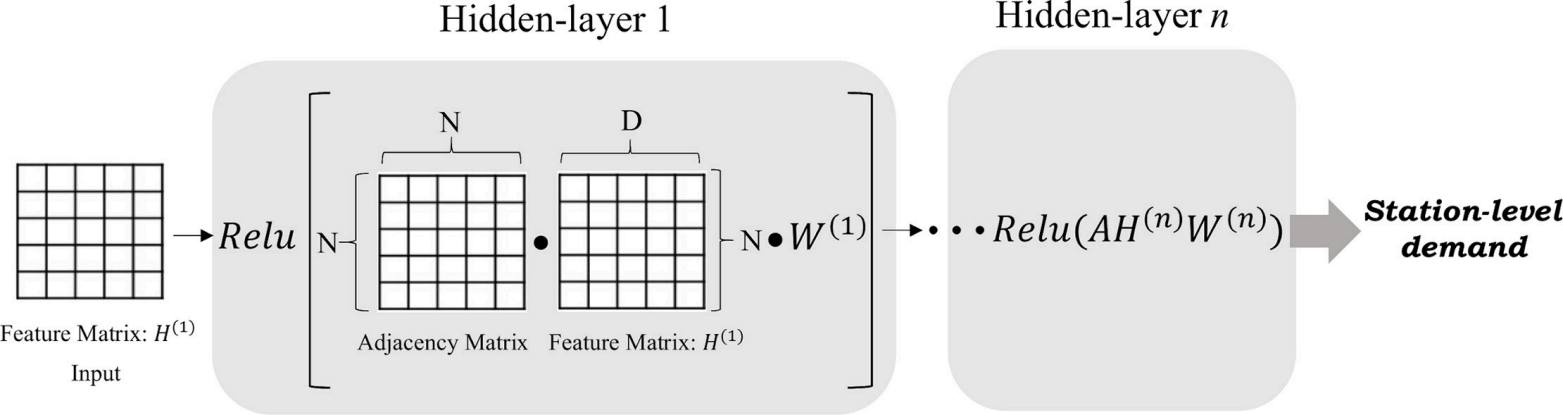
Architecture of GCNs.

Fig 2 shows the layer-wise propagation rule of the GCN. In all neural layers, feature matrix H is multiplied by adjacency matrix A. This matrix product allows dependencies among stations to project into every feature at each station. The output matrix of each layer becomes a new feature matrix in the next layer. When a new feature matrix enters the next layer, it is again multiplied by A. Thus, the connectivity between stations can be continuously injected to all features at each station. Finally, this process produces a station-level output, Z, the last feature vector, and station-level demand prediction can be performed by using this extracted feature.

$$H^{\left(l+1\right)}=f(H^{\left(l\right)},A)=\sigma (AH^{\left(l\right)}W^{\left(l\right)}),$$

where *H*^(*l*)^ is a feature matrix of the *l*^*th*^ layer, *W*^(*l*)^ is a weight matrix of the *l*^*th*^ layer, and *σ* denotes an activation function. However, A is not normalized. Therefore, the multiplication process completely changes the scale of the feature matrix. To adjust the scale, the adjacency matrix, A, should undergo a Laplacian normalization process [11], defined as

$$f(H^{\left(l\right)},A)=\sigma (D^{-\frac{1}{2}}AD^{\frac{1}{2}}H^{\left(l\right)}W^{\left(l\right)}),$$

where *D* is the diagonal matrix of *A*.

### Dependence among stations

The GCN’s performance depends on the relationship reflected between nodes. A representative description of the connection between nodes is in the form of an adjacency matrix. In this section, two spatial properties are used to define connectivity between stations. Correspondingly, we consider two types of adjacency matrices for quantifying the degree of connection.

(1) Inverse distance weight (IDW) matrix: GCN-IDW

The effect of geographical distance is one of the most basic spatial properties. If two stations are geographically approximate, they will be strongly connected, according to law of geography [6]. Thus, we use IDW to decay connectivity by spherical distances, using the stations’ latitudes and longitudes. The inverse distance weighting function is defined as

$${A^{IDW}}_{ij}=\left\{ \begin{array}{cc} \frac{\frac{1}{d_{ij}}}{\sum \frac{1}{d_{ij}}} & \left(i\neq j\right)\\ 1 & \left(i=j\right) \end{array}\right.,$$

where *A*^*IDW*^_*ij*_ is the element of distance weight matrix, and *d*_*ij*_ is the distance between stations *i* and *j*.

(2) Usage pattern (UP) matrix: GCN-UP

This approach makes use of the return and rent information from the bike usage records. The return–rent pattern indicates how much the rent record from the other stations (previous time) is relevant to the present demand of corresponding stations. Some stations, from which cyclists prefer to borrow, can easily experience a shortage of rentable bikes [18]. Because this increases dissatisfaction [19], the relation needs to be reflected in the model. Thus, we create a usage-pattern matrix based on return-rent trajectory data, described as

$${A^{UP}}_{ij}=\sum RR_{ij},$$

where *A*^*UP*^_*ij*_ is the element of usage pattern matrix, and *RR*_*ij*_ is the number of times borrowed from station *j* and returned to station *i* during the entire period.

### Various temporal dependence and fusion stage

**Fig 3:**
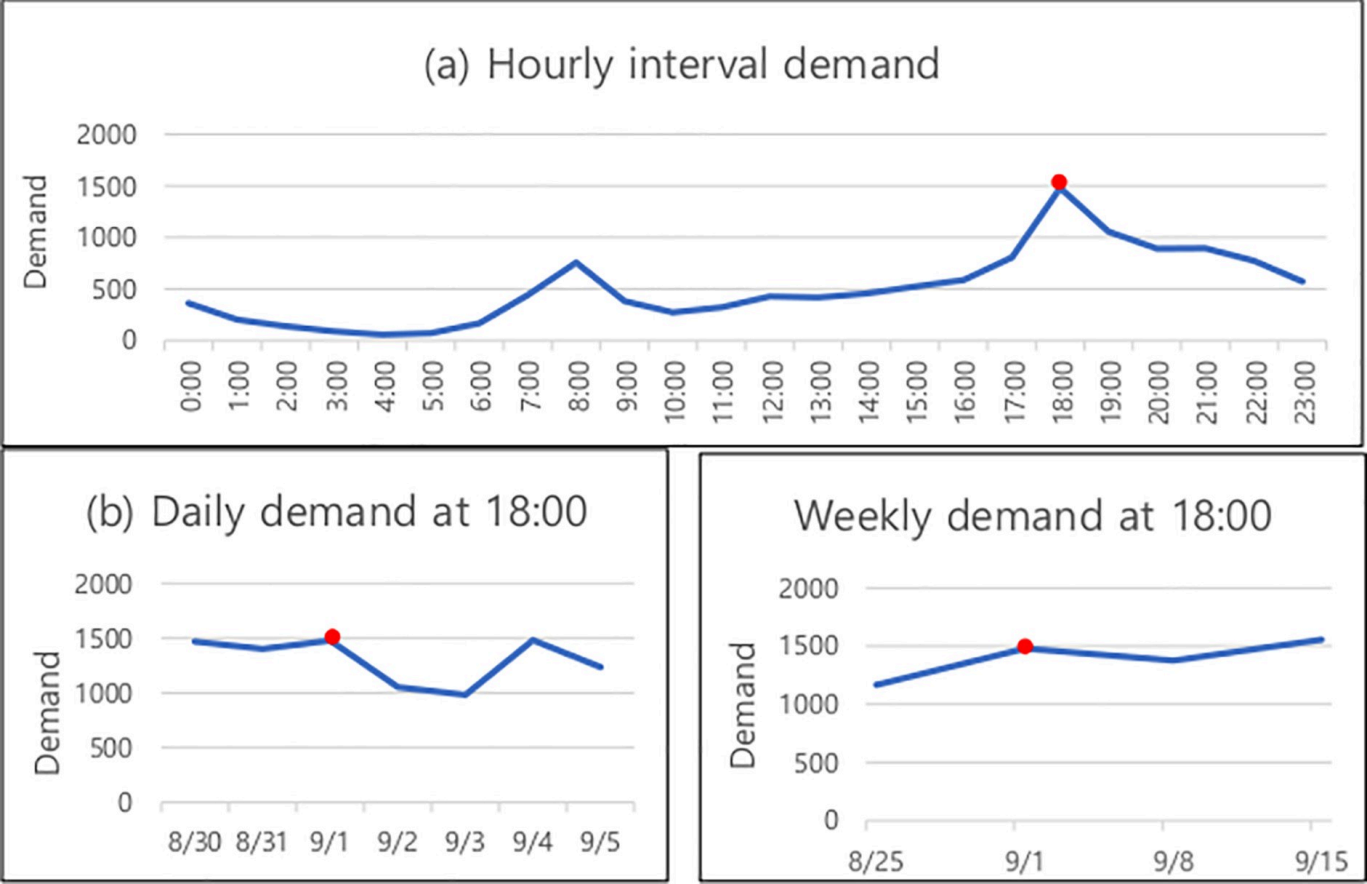
Various temporal patterns of bike demand. (a) Hourly demand during September 1, 2017; (b) daily demand during August 30-September 5, 2017; and (c) weekly demand for August 25 to September 1, 8, and 15, 2017, where the red point indicates September 1.

Bike data has various temporal patterns that can be used to predict demand [20]. Fig 3 shows temporal patterns of bike demand according to different time interval. Fig 3(A) shows the influence of the nearest time. Next time bike demand is close to that of peripheral usage, so unless there is a special event, this trend will be maintained. Meanwhile, bike usage also has the regular-patterns that are repeated at certain time intervals, as shown in Fig 3(B) and 3(C). Thus, sequential timestamp with time interval can be useful for predicting the next time demand. Reflecting these various temporal patterns can improve the prediction accuracy.

**Fig 4:**
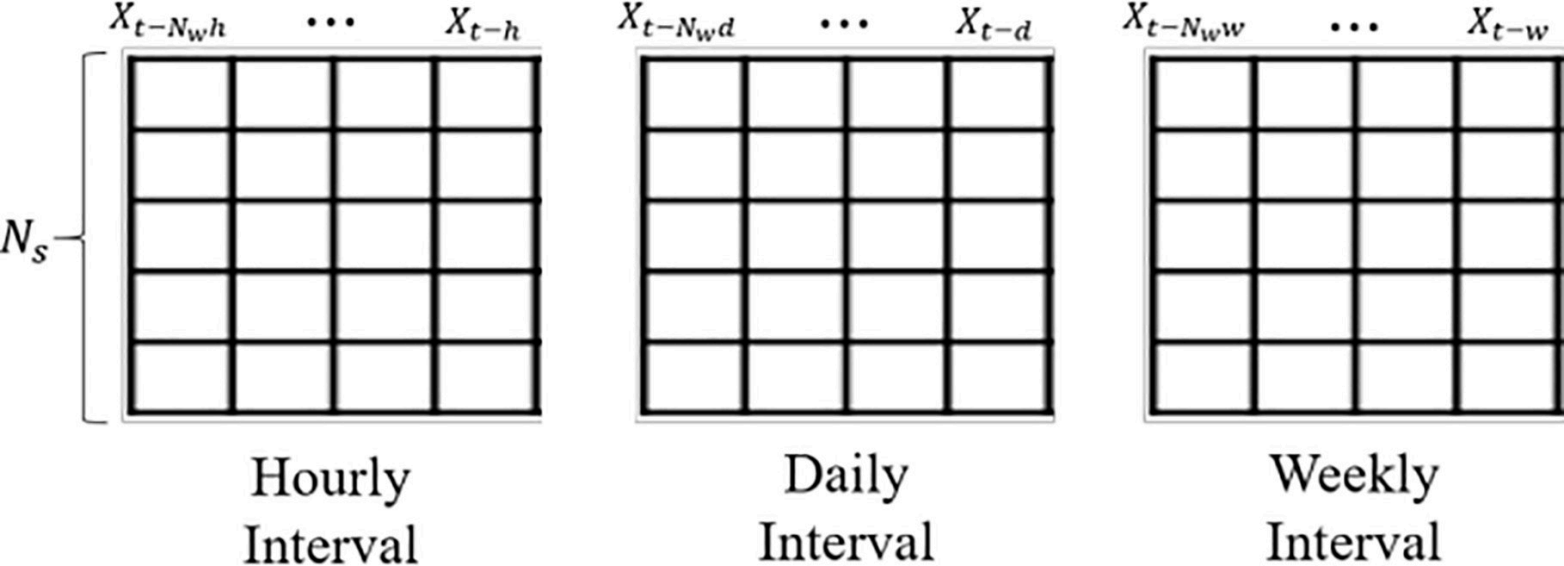
Feature matrix of hourly, daily, and weekly model (optimal *N*_*w*_ can be different for each model; *h*, *d*, and *w* represent 1 hour, 1 day, and 1 week, respectively).

However, it is very difficult to incorporate all properties in a single model [6]. To address this issue, we create three different GCN models: hourly, daily, and weekly. They differ in terms of time–feature matrix used. The hourly model is used to evaluate the aspect of the repeated daily pattern at the same hour. Thus, the feature matrix, stacked with hourly demand, is offered as shown in Fig 4. Because daily and weekly models are intended to consider daily or weekly patterns, feature matrices made of daily and weekly demand are organized respectively.

To determine each temporal pattern efficiently, we constructed three single GCN models (i.e., an hourly, daily, and weekly GCN model). Since each model used an individual temporal feature matrix and spatial adjacency matrix, the output feature reflected the spatio-temporal properties simultaneously. As we generate these three models having the same GCN structure. the outputs of each model are similarly denoted as

$${H_{h}}^{\left(Z\right)}=f({H_{h}}^{\left(0\right)},A),$$

$${H_{d}}^{\left(Z\right)}=f({H_{d}}^{\left(0\right)},A),$$

$${H_{w}}^{\left(Z\right)}=f({H_{w}}^{\left(0\right)},A),$$

where f (H, A) is the GCN propagation rule, *H*_._^(0)^ is the first feature matrix of each model, and *H*_._^(*Z*)^ is the output feature of each model. To determine the various temporal patterns together, we combined all output features extracted from each model and fused them into a FCL. The input feature vector of the FCL was denoted as *H*_*c*_^(*z*)^ = {*H*_*h*_^(*z*)^; *H*_*d*_^(*z*)^; *H*_*w*_^(*z*)^}, which concatenated all the output features.

### Global variables

One of the biggest influences on bike usage is the cycling environment [21]. Radical changes to the cycling environment are big obstacles to improving prediction accuracy [22]. Thus, capturing the change in meteorological conditions was beneficial for predicting bike demand. We considered these meta features as global variables because of their broad influence, which worked equally for all stations. In this study, we obtained two meta datasets, namely weather and weekday/weekend, as global variables for the corresponding time *t*.

**Fig 5:**
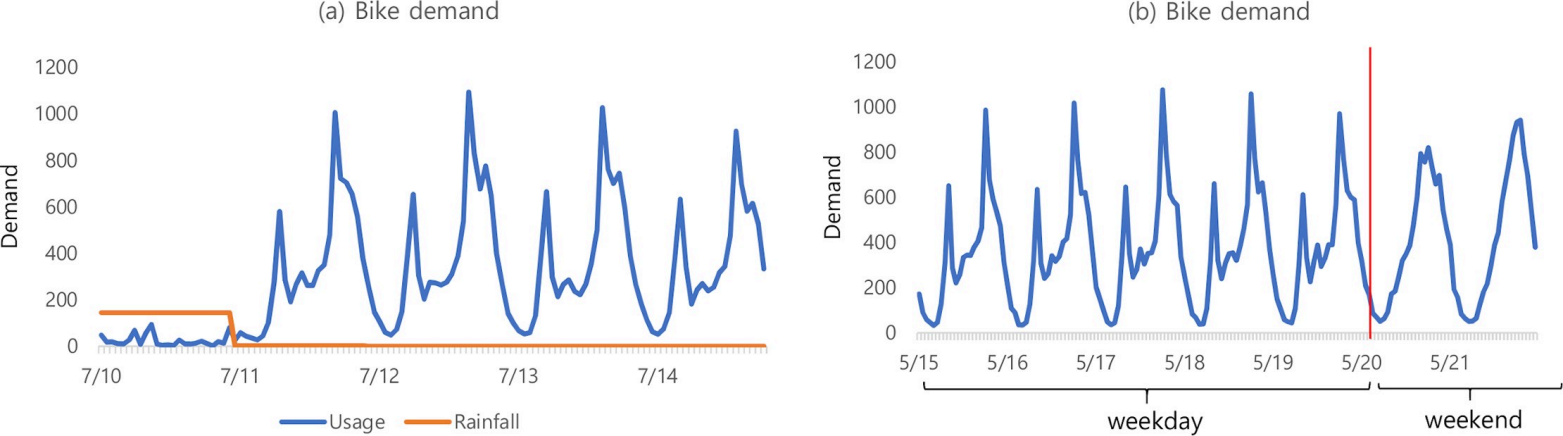
Total demand of all stations depending on global variables: (a) Bike usage according to rainfall (b) Bike usage according to weekday or weekend.

In various cycling environments, weather has the greatest influence on usage [23], and rainfall has the strongest impact on demand [24]. It is less enjoyable to cycle in the rain, and also increases the risk of traffic accidents. As shown in Fig 5(A), bike usage at rainy days would be much less than usual. We collect hourly precipitation data from the meteorological agency and estimate the influence of precipitation. The impact of precipitation at every station is assumed to be the same. Another global variable that we considered was different usage patterns during the weekdays and weekends. Bike usage is closely related to life patterns [25]; in particular, the different patterns between weekdays and weekends can have a significant impact on identifying the bike usage distribution. Fig 5(B) shows high total bike usage during weekday commutes and high bike usage in late afternoons on weekends. Thus, we reflected the different influences depending on whether the corresponding time *t* was during the weekday or weekend.

We transform these global variables into dummy variables, merging them with the output of FCL. Finally, the predicted value at time t is defined as

$${\hat{y}}_{t}=W_{c}*{H_{c}}^{\left(z\right)}+W_{w}*G_{w}+W_{d}*G_{d},$$

where *W*_._ is the parameter of output and global variables, with respect to the degree of rainfall *G*_*w*_ and the weekday/weekend *G*_*d*_. We use the mean squared error (MSE) as a loss function:

$$\frac{1}{T*N_{s}}\sum_{i}^{T}\sum_{j}^{N_{s}}{{(\hat{y}}_{ij}-y_{ij})}^{2},$$

where T is number of hours, ${\hat{y}}_{ij}$ is value of prediction, and *y*_*ij*_ is value of hourly usage per station. Learning is progressed to minimize the loss function.

## Empirical study

### Data description

In this study, we applied our model to a real-world dataset provided from the Seoul bike sharing system, “ddareungi”, from Korea Public Data Portal. It has over 70-million bike records, spanning from September 19^th^, 2015 to December 31^st^, 2017. The original data are divided into two sections: bike transaction data (i.e., user id, rent/return time, rent/return station name and station number) and station data (i.e., station name, station number, station address, number of total usable bike of each station, latitude and longitude).

There were nearly 100 stations at the beginning of 2016, and demand was very sparse. However, the number of stations increased explosively in 2017 until it has exceeded 1000, and bike-sharing demand grew. Thus, we only selected the data after 2017, and we used stations that existed since the beginning of 2017, having more than 1 average usage per hour. As a result, we used 174 stations and about 12 million bike record data.

### Data preprocessing

For each station, 6,552 hourly demands were prepared as the total sequential dataset. The standard normalization was applied to standardize the bike demand per station. The first 5,832 hourly demands (i.e., 8 months) were used as the training set and the last 720 hourly demands (in September) were prepared as the test set.

To transform the continuous demand into a graphical structure, three feature matrixes *H ∈ R*^*Ns*×*Nw*^, where *N*_*s*_ and *N*_*w*_ indicated 174 stations and various temporal window sizes for each GCN model, respectively, were constructed, and the current demands of whole stations were converted into the corresponding target vector *y*_*t*_ *∈ R*^*Ns*^. For each GCN model, the feature matrix consisted of the previous demand with hourly, daily, and weekly time intervals; the window size could be different to predict the same target vector.

### Experimental Settings

#### GCN model

For the hyperparameter optimization, a subset of hyperparameters needed to be selected before the learning process. In machine learning systems, hyperparameters have a significant influence on determining the optimal model that minimizes the predefined loss function. To conduct the hyperparameter optimization, we conducted a grid search that replaced the exhaustive enumeration of all combinations with a manually specified subset of the hyperparameter space. The performance based on the chosen hyperparameters was measured using the test set.

We had three different GCN models, namely an hourly, daily, and weekly model. Each GCN model had a different number of features based on its window size. Table 1 summarizes the various subsets of the window sizes that we considered. Through trial and error, we inferred that window sizes smaller than 3 or larger than 5 increased the MSE result because of a lack of information or because of overfitting. The optimal window size for the hourly model had an input dataset of *t*-4, *t*-3, *t*-2, and *t*-1 for predicting demand from time *t*, and the daily and weekly models forecasted the demand at time *t* with the demands from the previous 1–3 days as well as those from the previous 1–3 weeks at time *t*, respectively.

**Table 1:** Selecting the optimal window size by grid search.

Number of hours	Number of days	Number of weeks	Result (MSE)
3	3	3	5.23
4	4	4	5.50
5	5	5	5.62
3	3	2	5.34
3	4	3	5.17
**⋮**			
**4**	**3**	**3**	**5.12**
4	4	3	5.20

Our GCN models were constructed using Python and Tensorflow. Note that all the GCN models had the same internal structure, which was composed of two hidden layers. We imposed various numbers of nodes to each layer and determined the optimal values as 16 and 32 nodes, respectively. Between the layers, rectified linear unit (ReLU) was used as the activation function to perform non-linear transformation and the dropout technique was used to reduce the overfitting problem. For stochastic learning and backpropagation, Adam was chosen as our optimization algorithm and the learning rate was 10^−3^. Total 500 epochs were repeatedly executed and the early stopping technique was employed for efficient convergence. The results of the hyperparameter optimization are presented in Table 2.

**Table 2:** Description of hyperparameter values.

Hyperparameter	Value	Hyperparameter	Value
Window size (hour/day/week)	4/3/3	Number of hidden nodes	16/32
Activation function	*ReLU*	Dropout rate	0.5
Learning rate	0.001	Number of epochs	500

#### Baselines

The proposed GCN model is able to provide accurate prediction of bike demand. To verify superiority of our proposed model, we considered four regression based prediction models: autoregressive integrated moving average (ARIMA), extreme gradient boosting algorithm (XGBoost), recurrent neural network (RNN), long short-term model (LSTM), and two different methods in the transportation domain, namely the GBM approach [14] and GCN approach based on a data-driven graph filter (GCN-DDGF) [1]. Moreover, we built four types of conditional GCN models to test contribution of various temporal patterns as well as global variables. Each conditional GCN reflects one of the temporal patterns or global variables. In addition, our proposed GCN models, GCN-UP and GCN-IDW were also implemented for evaluation.

#### ARIMA

We use autoregressive integrated moving average (ARIMA) which can reflect periodicity and seasonality in the time-series [26]. Because ARIMA was the representative model in the time-series domain, many studies on the transportation domain have used it as a baseline [27–28, 6, 9]. In the ARIMA model, p, d, and q are represented, where p is the order of the autoregressive part, d is number of times data series is differenced, and q is the order of the moving average part. Because the p, d, and q differ according to dataset, we used the parameters most suitable for each station.

#### XGBoost

We consider the extreme gradient boosting algorithm (XGBoost) as one of our baselines. Basically, XGBoost belongs to ensemble tree learning algorithm, but it estimates the different weight of parallel decision tree [29]. It normally uses classification and regression tree (CART) and update weights of trees by gradient boosting algorithm. Moreover, it develops model performance and computational speed by novel distributed techniques such as weighted quantile sketch, novel sparsity-aware algorithm and cache-aware block structure [29]. Owing to its powerful performance, XGBoost was recorded as one of the best models in the 2014 Kaggle Bike Sharing Demand Prediction competition. We have constructed a dataset of temporal window size 10 where 10 hourly sequential usage is used to predict the following value and the target value is the following demand. We employed CART as the decision tree for the regression and set the number of trees to 10, maximum depth of each tree to 3 and learning rate to 10^−2^ through trial and error.

#### RNN & LSTM

We use the recurrent neural network (RNN) that is effective neural network model for time-series data. RNN can handle the sequential information because it has the feedback connection from input to output by preceding layers [30]. However, since RNN has gradient vanishing problem that represents shrinking gradient during back propagation, traditional RNN is not appropriate for capturing long-term dependencies [31]. To avoid this issue, long short-term models (LSTM), our last baseline model, has been used as alternative to RNN. The LSTM is RNN based model that can discover the long relationship of time-series data [32]. Unlike RNN, LSTM has the four gates such as input gate, forget gate, cell and output gate which are introduced in Hochreiter & Schmidhuber [32], and these gates weaken decreasing tendency of gradient during long-term back propagation. We constructed an RNN model with 3 RNN layers and temporal window size of 4, and an LSTM model with 3 LSTM layers and temporal window size of 3. We used 10^−2^ learning rate and repeated learning process as much as 1,000 epochs for both models.

#### Comparison with related approaches

The comparison of the performance of the prediction of bike demand using different methods was also included in the experiment results. We chose two related methods, namely the GBM approach of Regue & Recker [14] and the GCN-DDGF approach of Lin et al. [1]. The GBM is a decision tree-based algorithm that has an internalized variable selection effect that is sensitive to variation in explanatory features and insensitive to outliers. For GBM implementation, we utilized the *gbm* package in R developed by Ridgeway [33]. The learning rate and number of iterations, which are regularization parameters in the GBM, were set as 10^−2^ and 500, respectively.

The GCN-DDGF approach of Lin et al. [1] is similar to our GCN approach, but they parameterized the adjacency matrix to determine the predefined loss function. In a graphical analysis, prior knowledge is required to construct the adjacency matrix, and the performance depends on the extracted component. However, the parameterized adjacency matrix does not require prior knowledge and provides stable performance for any process. We used the same GCN structure as that of our model but imposed the parameterized adjacency matrix to perform GCN-DDGF.

#### Conditional graph convolutional neural network models

The proposed novel GCN model considered various temporal patterns and global variables as the different factors to predict the next hour demand, and graphical convolution was performed on these indexes. However, it was not clarified whether GCN can extract useful information that is not only from spatial characteristics. Therefore, we organized four additional GCN models based on the following separated information: hourly information (HM), daily information (DM), weekly information (WM), and discarding global information (DGM). We compared their performances with those of the GCN-UP and GCN-IDW to demonstrate the superiority of using incorporated information.

#### Evaluation metric

In order to compare all these baselines to ours, we use both the MSE and Pearson correlation as evaluation metrics. The MSE is used to measure the average squared errors between the actual and predicted values. Meanwhile, continuous demand in multiple locations at different time points is represented by spatio-temporal distributions, which is also important to predict spatio-temporal distributions in the transportation domain [34]. Thus, the average Pearson correlation (APC) was utilized as one of our evaluation metrics to compare the similarity between actual and predicted distributions. The APC can be expressed as follows:

$$APC=\frac{1}{N_{s}}\sum_{s=1}^{N}\frac{cov({\hat{y}}_{s},y_{s})}{\sigma_{{\hat{y}}_{s}}\sigma_{y_{s}}}$$

where ${\hat{y}}_{s}$ and *y*_*s*_ represent the predicted demand and actual demand at station *s* over the time period, respectively. *cov* represents covariance while σ represents standard deviation.

### Experiment results

#### Comparison result with evaluation metrics

Comprehensive comparison among the results of bike demand prediction are shown in Table 3. For a robust comparison, we conducted experiments for two test periods, August and September. However, as the demand in August was far less than that in September, there was no significant difference among model performances in the August test set. Our proposed model has the outstanding performance compared to other baseline models. This indicates that the simultaneous consideration of spatio-temporal properties can enhance the proposed GCN models (i.e., GCN-IDW and GCN-UP) by reflecting implied attributes in bike demand, and that GCN models can extract useful features from incorporated information of various temporal patterns and global variables to improve prediction performances.

**Table 3:** Performance comparison of different approaches.

	August		September	
Models	MSE	APC	MSE	APC
**ARIMA**	4.96	0.38	8.5	0.3
**XGBoost**	1.92	0.78	7.24	0.59
**RNN**	1.36	0.82	6.18	0.63
**LSTM**	1.36	0.82	6.03	0.64
**GBM [14]**	2.12	0.78	7.56	0.58
**GCN-DDGF [1]**	1.16	**0.84**	5.62	0.67
**GCN-HM**	1.86	0.79	7.61	0.49
**GCN-DM**	1.96	0.79	7.92	0.44
**GCN-WM**	1.96	0.8	8.22	0.34
**GCN-DGM**	1.88	0.8	6.81	0.59
**GCN-IDW**	1.25	0.83	5.65	0.67
**GCN-UP**	**1.14**	0.83	**5.15**	**0.69**

Between our two GCN models, the GCN-UP had better performance than GCN-IDW for the test set from both September and August. From this result, it could be considered that the usage pattern of the user was more related to demand than to the distance between stations. The GCN-UP model was followed by the GCN-DDGF model, which had better performance than the GCN-IDW model. While the GCN-DDGF model can capture hidden heterogenous pairwise correlations between stations [1], it has a risk of overfitting and weakness to time complexity due to the increased number of parameters. The number of parameters in the GCN-DDGF model increased exponentially with the number of stations. In addition, the proposed GCN models in this study coped with additional factors related to bike demand. These were why the GCN-DDGF model did not always perform better than when using a predefined adjacency matrix.

The next best performance is LSTM, but followed by RNN with a very slight difference. This indicates that both models can be limited only with the temporal dependencies while reflecting both spatial and temporal properties makes our model outperform than RNN and LSTM. The LSTM and RNN were followed by the GCN-DGM model, which had the best performance among the conditional GCM models. The impact of global variables on forecasting bike demand was superior to capturing various temporal patterns. The XGBoost and GBM performed better than the other conditional GCN models. The XGBoost is known for its excellent performance, and the GBM has a similar structure to that of XGBoost, but they were not designed to consider the spatial or temporal dependencies of bike demand. Therefore, their performances were very poor in our time-series regression task. The GCN-HM, GCN-DM, and GCN-WM models followed the XGBoost and GBM sequentially, and ARIMA had the worst prediction power in all the cases. Unlike the RNN or LSTM, ARIMA reflects only the independent temporal pattern of each station. It also shows the capturing complexity of temporal patterns and has a significant influence on predicting the transportation system.

#### Prediction power of station-level

**Fig 6:**
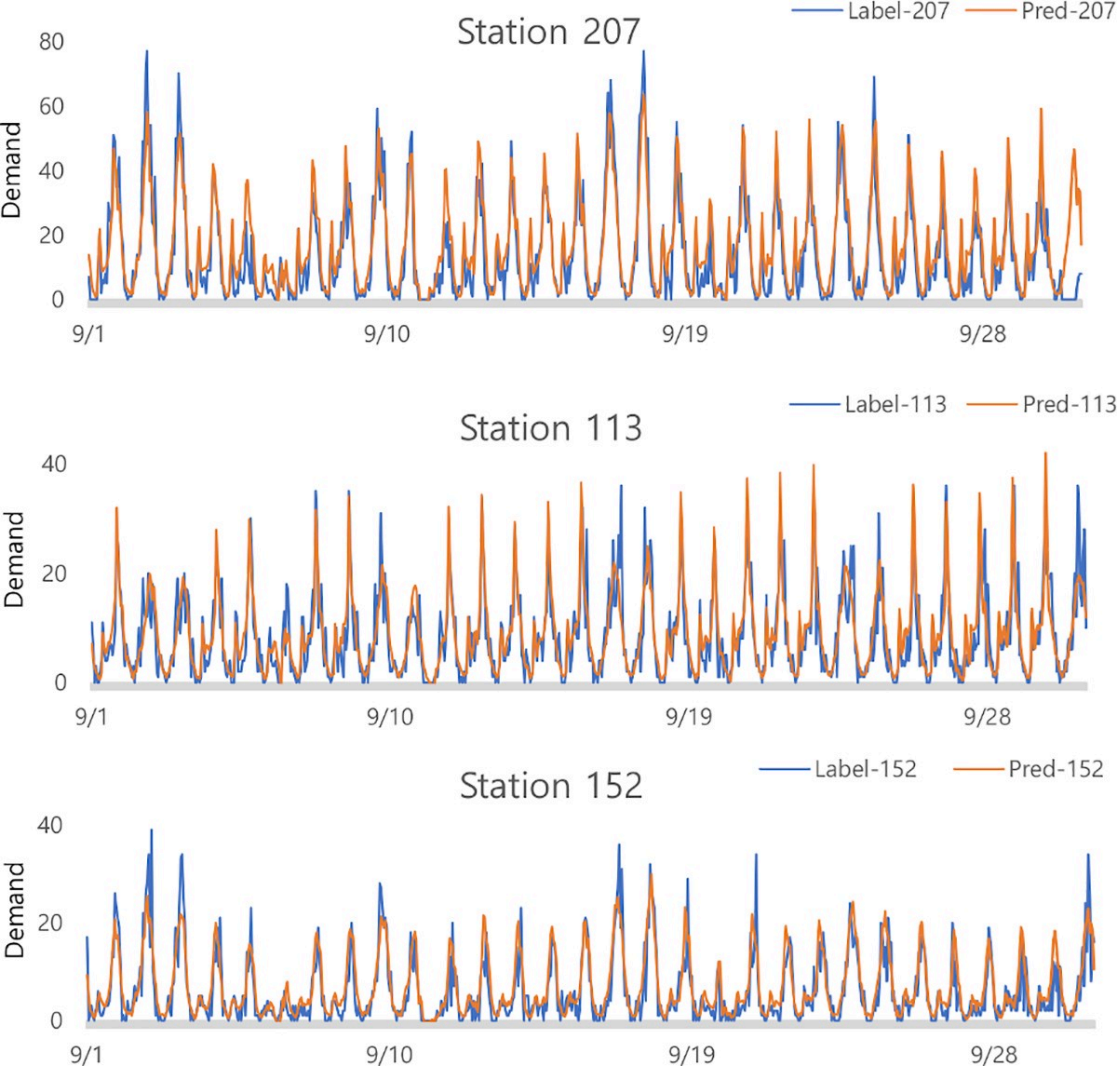
Plot comparing the predicted value with the actual value of the three busiest stations: Blue line is value of label and orange line is value of prediction.

The purpose of our study was to increase the prediction accuracy for each station. The predicted value of each station can be poor, even if the MSE is small for the total demand. To demonstrate the forecasting power at the station level, we presented plots to show the difference between real demand and predicted values, as in Fig 6. We selected the top three busiest stations, which recorded the highest total bike usage among all stations. Results show that our model predicted the actual demand well, and the performance of our model was robust to sudden environmental changes. This is because our model well reflects the spatio–temporal properties as well as the influence of global variables. Thus, the performance of our model is robust to the dynamic changes in the cycling environment.

#### Multi-step ahead prediction

Generally, time-series forecasting predicts the observation at the next time step. However, with the same historical data and trained model, we can predict multi step-ahead values in advance. This multi-step ahead prediction shows the robustness of the prediction power, regardless of the forecasting horizon size. The traditional method of performing multi-step ahead prediction is through a direct multi-step forecast strategy. The direct method involves developing a separate model with an independent training process as the step size changes [35].

**Fig 7:**
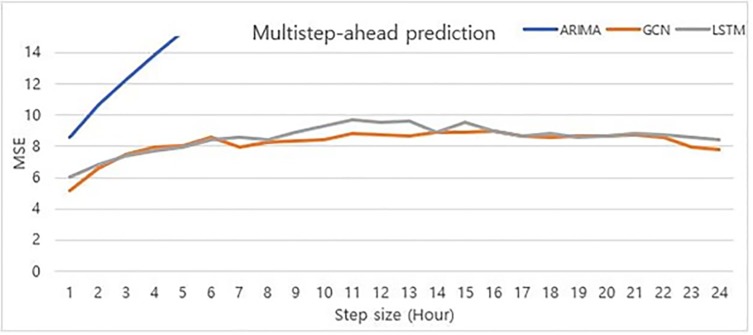
Prediction power of multi-step ahead for each model.

Fig 7 shows the comparison results of our model to baselines. As the step size grows, the MSE of ARIMA model increases rapidly. Thus, we only display the MSE of ARIMA up to step-size 3. However, our model and LSTM, which are deep learning models with enough complexity, enjoyed stabilized performance irrespective of the forecasting step-size. From steps 1 to 6, their performances were nearly equal; however, after step 6, the difference in performance increased. Our GCN model had better performance compared to the other time-series baselines. Moreover, the MSE of ARIMA and LSTM continuously increased, while our GCN model showed a decreasing trend in MSE in step 7 and 12. Thus, it was demonstrated that our GCN model performed best in terms of both prediction and stabilization.

## Conclusion

This paper proposed a GCN framework for hourly demand prediction of bike usage at the station level. We used two graph structures (i.e., GCN–IDW and GCN–UP) to reflect different spatial properties and compare performance. To consider various temporal patterns simultaneously, we trained three individual GCN models with hourly, daily, and weekly patterns, respectively. Additionally, the influences of rainfall and weekday/weekend were considered as global variables. Thus, the predictive power of our model was robust to sudden changes in the cycling environment. Experiments show that our model outperformed the other prediction models, and the historical usage pattern was a more suitable spatial property of demand than geographical distance. Thus, our study contributes to reducing the supply–demand imbalance.

However, GCN can only extract features from a predefined graph structure. The appearance of a new station cannot be reflected in the analysis. If GCN can flexibly cope with a new station, it will be able to reflect changing relationships in real time. Furthermore, considering more variables such as sudden events or other weather can make more accurate prediction. Moreover, current proposed models can also be utilized to other transportation problem which is also threatened by supply-demand imbalance problem. These issues should be investigated further.
