# Correction: Graph convolutional network approach applied to predict hourly bike-sharing demands considering spatial, temporal, and global effects

**DOI:** 10.1371/journal.pone.0266221

**Published:** 2022-03-24

**Authors:** Tae San Kim, Won Kyung Lee, So Young Sohn

The following information is missing from the Data Availability Statement: The meteorological data underlying this study are available from https://www.weather.go.kr/w/obs-climate/land/past-obs/obs-by-day.do?stn=108&yy=2016&mm=4&obs=1.

The following information is missing from the Funding statement: In addition to 2019R1H1A207, this research was partly supported by ’Geospatial Big Data Management, Analysis and Service Platform Technology Development’ project for the MOLIT (The Ministry of Land, Infrastructure and Transport), Korea, under the national spatial information research program supervised by the KAIA (Korea Agency for Infrastructure Technology Advancement) (18NSIP-B081011-05). This work is an updated and extended version of the early local presentation made at KOREA INFORMATION SCIENCE SOCIETY conference by the same authors: "Bike Sharing Demands Prediction based on GCN", Proceedings of the KISS Conference p832-834, 2018.
